# Vitamin E and Non-Communicable Diseases: A Review

**DOI:** 10.3390/biomedicines10102473

**Published:** 2022-10-03

**Authors:** Giulia Ciarcià, Simone Bianchi, Barbara Tomasello, Rosaria Acquaviva, Giuseppe Antonio Malfa, Irina Naletova, Alfonsina La Mantia, Claudia Di Giacomo

**Affiliations:** 1 Department of Drug and Health Sciences, University of Catania, Viale A. Doria 6, 95125 Catania, Italy; 2 Research Center on Nutraceuticals and Health Products (CERNUT), University of Catania, Viale A. Doria 6, 95125 Catania, Italy; 3 Nacture S.r.l., Spin-Off University of Catania, 95125 Catania, Italy; 4 Institute of Crystallography, Research National Council, 95126 Catania, Italy

**Keywords:** Vitamin E, tocopherols, tocotrienols, antioxidant, anti-inflammatory, diabetes, asthma, cardiovascular diseases, cancer

## Abstract

Vitamin E, a nutrient found in several foods, comprises eight lipophilic vitamers, the α-, β-, γ- and δ-tocopherols and the α-, β-, γ- and δ-tocotrienols. This vitamin is capable of exerting antioxidant and anti-inflammatory activities, and acting as immunomodulators. Despite these well-known biological activities, the findings regarding the ability of vitamin E and its serum metabolites to prevent and/or control chronic disease are often conflicting and inconsistent. In this review, we have described the metabolism of vitamin E and its interaction with the gut microbiota, considering that these factors may be partially responsible for the divergent results obtained. In addition, we focused on the correlations between vitamin E serum levels, dietary intake and/or supplementation, and the main non-communicable diseases, including diabetes mellitus, asthma, cardiovascular diseases, and the four most common cancers (breast cancer, lung cancer, colorectal cancer, and prostate cancer) with the intention of providing an overview of its health effects in the non-communicable-diseases prevention.

## 1. Introduction

Vitamin E was first recognized as an essential molecule by Evans and Bishop 100 years ago and was originally named “factor X” or “anti-sterility factor” [1,2]. To celebrate the centennial of the discovery of vitamin E, some research related to vitamin E, its serum metabolites, its roles in preventing and/or its therapeutic roles in the most common chronic diseases and the possible correlation with gut microbiota are discussed. Vitamin E, a fat-soluble antioxidant, is mainly involved in the inhibition of biological membranes oxidation. Moreover, its beneficial health effects can also be related to its interaction with receptors, transporters, enzymes, and transcription factors. To exert its regulatory effects, vitamin E must be effectively absorbed when introduced with food and then distributed throughout the body; all these pharmacokinetic features are mainly influenced by its hydrophobic properties [2,3].

**Figure 1:**
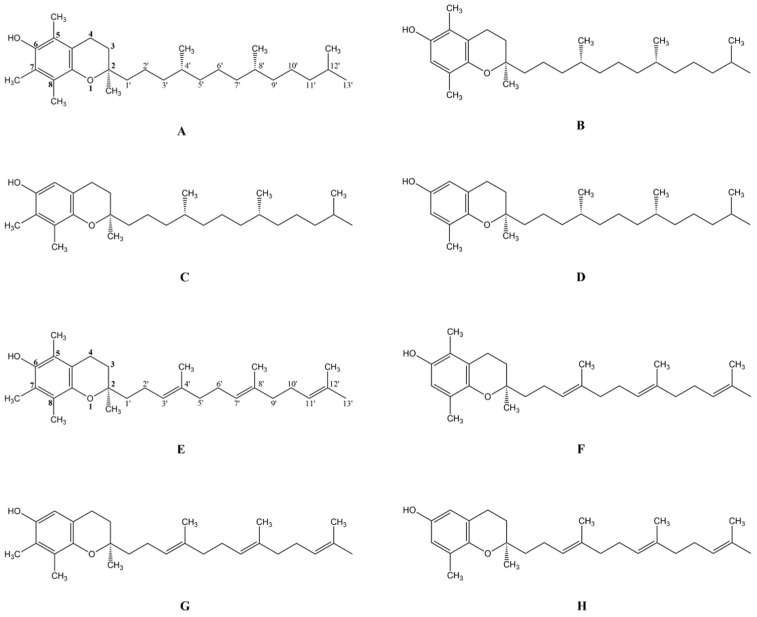
Naturally occurring tocopherols (**A**–**D**) and tocotrienols (**E**–**H**): α-tocopherol (**A**); β-tocopherol (**B**); γ-tocopherol (**C**); δ-tocopherol (**D**); α-tocotrienol (**E**); β-tocotrienol (**F**); γ-tocotrienol (**G**); δ-tocotrienol (**H**).

The vitamin E family has eight lipophilic vitamers: α-tocopherol (A), β-tocopherol (B), γ-tocopherol (C), δ-tocopherol (D) (αT, βT, γT, δT) and α-tocotrienol (E), β-tocotrienol (F), γ-tocotrienol (G), δ-tocotrienol (H) (αTE, βTE, γTE, δTE (Figure 1). Tocopherols and tocotrienols share the same basic chemical structure, which consists of a chromanol ring, whose phenolic group can donate electrons for scavenging mainly lipid peroxyl radicals. In detail, at carbon 2 of chromanol ring, tocopherols have a saturated side chain while tocotrienols have a three unsaturated side chain. Each analogous α-, β-, γ-, and δ- differs at the 5- or 7-position on the chromanol ring for a hydrogen (-H) or a methyl (-CH3) group. The natural forms of tocopherols have three chiral carbons in the R-conformation at the 2, 4′ and 8′-position, and tocotrienols exist naturally as 2R stereoisomers. Conversely, synthetic forms of αT are a blend of R or S-stereoisomers at the position 2, 4′ and 8′ [2,4].

## 2. Metabolism

To clarify how vitamin E acts, it is useful to understand its metabolism and dietary sources. It is well known that a high intake of these vitamin E isoforms can be optimally achieved through the consumption of plant seeds and oils, rich in certain types of dietary fatty acids. Generally, tocopherols are found in nuts and vegetable oils, and barley oat, palm oil, rice bran, rye, and wheat germ mainly contain tocotrienols. Furthermore, vitamin E isomers naturally occur in other daily foods such as fruits, eggs, cheese, and seafood [4].

Intake of vitamin E from natural sources results in the formation of micelles with bile acids, cholesterol, phospholipid, and triacylglycerol in the intestinal lumen. Similar to cholesterol, vitamin E is also found in almost all organs and cell types and approximately three grams of vitamin E are stored in the body of a healthy adult. The main reservoirs of vitamin E are in subcutaneous fat, muscles, and liver, however its concentration per gram of tissue is high in adipose tissue, adrenal glands, pituitary glands, and testes [5,6,7].

Similar to other lipids, vitamin E forms chylomicrons with cholesterol, phospholipids, and triacylglycerol in the lacteal vessel. Chylomicron containing vitamin E circulates via the lymphatic system (reaching to peripheral tissues) to the bloodstream via the thoracic duct. Vitamin E contained in chylomicrons then reaches the liver before it is subsequently taken up by very low-density lipoprotein (VLDL) and released into the bloodstream [4,5,6,7].

Tocopherols and tocotrienols have similar antioxidant activities, but markedly different bioavailability. The αT represents the predominant form of vitamin E in tissues and it has much higher bioavailability than other vitamin E forms. Two proteins play critical roles in controlling metabolism of vitamin E in the liver, in particular, the tocopherol transport protein (αTTP), a cytosolic protein of about 30 kDa with high affinity for αT, and vitamin E ω-hydroxylase (CYP4F2). Some works have revealed that only αT is sequestered in the body by the hepatic αTTP, whereas excess αT, and the other seven analogues showing low affinity for αTTP, are not retained but are rapidly metabolized by phase I metabolism (catabolism and side chain shortening) and phase II metabolism (sulfation and glucuronidation), and finally excreted via faeces and urine. Unlike the in vivo dynamics of αT storage in various organs, the distribution of tocotrienol is tissue-specific (e.g., brain and adipose tissue) probably because its distribution occurs via the lymphatic system instead of through the bloodstream as for αT [5,6,7].

The other vitamers retained in the liver are metabolized in peroxisomes and mitochondria by CYP4F2, which catalyses the enzymatic hydroxylation reaction of a terminal methyl group of the sidechain by stepwise removal of 2 or 3 carbon moieties. The process results in the shortening of the sidechain up to the formation of 2-(2′-carboxy-ethyl)-6-hydroxychromans (CEHCs), which are bioactive vitamin E metabolites. Indeed, accumulating evidence indicates that specific metabolites, including CEHCs and 13′-carboxychromanol (13′-COOH), have similar biological activities compared to their precursors [6,7]. Among different isoenzymes of the cytochrome P-450 family, CYP4F2 is proposed to possess tocopherol-α-hydroxylase activity, but there is the possibility that other forms may play a role in this metabolism. In addition, cytochrome P450 (CYP) catalyses the omega-hydroxylation, followed by the beta-oxidation of the side chain in various vitamin E isomers [6,7].

It has been reported that vitamin E metabolites of possible interest may also be produced through a non-enzymatic oxidation. It is well known that vitamin E represents a physiological chain-breaker of lipid peroxidation reactions by donation of an H-atom to hydroperoxyl radicals formed during lipid peroxidation of polyunsaturated lipids. The tocopheroxyl radical produced during the one-electron oxidation of αT, α-tocopheronolactone hydroquinone (α-TLHQ), has been described to further react with lipid peroxides, leading to the formation of 8-substituted tocopherones. The analysis of these compounds was used to give a measure of lipid peroxidation in cellular membranes. Early in vitro and in vivo experiments have demonstrated that CEHC metabolites may also potentially exert a biologically relevant antioxidant function through mechanisms and reaction intermediates like vitamin E homologues [2,4,5,6,7]. In conclusion, this evidence suggests that non-enzymatic metabolites could be formed from the free radical-dependent oxidation of vitamin E, i.e., during its antioxidant function.

## 3. Serum Metabolites

The serum concentrations of the metabolites are in the nanomolar scale, and their biological activity seems to show new insights toward the function of vitamin E as a vitamin and its protective effects. All vitamin E vitamers are susceptible to oxidation by CYP4F2, chiefly those with different methylation patterns of the 6-hydroxychromanol ring, or those with one or more double bonds within the side chain. The ω-oxidized tocopherols and tocotrienols (namely 13-hydroxychromanols) are pictured as phase-I metabolic intermediates that prevent accumulation of the vitamin. Oxidation of the hydroxyl group leads to the long-chain metabolites (LCMs) 13’-COOHs, that are further β-oxidized to medium- and short-chain metabolites. As with other long-chain lipids, β-oxidation of LCMs occurs in the peroxisomes yielding 11’-COOH and 9’-COOH LCMs. Further β-oxidation steps take place within the mitochondrial matrix; here, intermediate-chain metabolites (ICMs, 7’-COOH, and 5’-COOH) and short-chain metabolites 3’-COOH (or carboxy-ethyl-hydroxy-chromanol [CEHC]) are formed. In general, all metabolites were found in free form or as sulphates or glucuronides. Methylation patterns of the chromanol ring are the same as for the parental vitamers, thereby forming four different CEHC homologues identified with the Greek letters, α, β, γ and δT, which are the major vitamin E compounds found in human blood and tissues [3,5,8].

Specifically, 2,5,7,8-tetramethyl-2-(2’-carboxyethyl)-6-hydroxychroman (α-CEHC) and 2,7,8-trimethyl-2-(2’-carboxyethyl)-6-hydroxychroman (γ-CEHC) are the main metabolites analysed [3,8,9]. Conversely, ICMs, such as 11′-, 9′-, 7′- and 5-carboxychromanols, usually are found at very low concentrations in biological fluids. Studies have also shown that α-CEHC may be formed from excess αT that was not sorted by the αTTP [5,9]. Alteration of the CYP4F14 gene, an orthologue of the human CYP4F2 gene, was responsible for an increase in ω-1 and ω-2 oxidation compounds and 11’-hydroxy-tocopherol (11’-OH: γ- and δ-11’-OH) both in mice and humans. Recently, some studies have shown that CYP4F2 catalysed the ω-hydroxylation of α-tocopheryl quinone [3,5,7].

What we aim to highlight is how and to what extent the serum metabolites have a beneficial effect on pathologies. Indeed, studies have widely demonstrated the biological activity of vitamin E metabolites as bioactive molecules with anti-inflammatory, anti-proliferative, and anti-atherogenic properties. Moreover, these metabolites were investigated for their effects on apoptotic signalling and lipid metabolism. Concomitantly, δT-13′-OH, δT-13′-COOH, and δTE-13′-COOH can exert potent in vitro antioxidant activities by blocking lipid peroxidation. Notably, the LCMs have shown biological activities in the nanomolar range compared to their metabolic precursors, so they likely play key roles in γT, δT, and tocotrienols-mediated anti-inflammatory and disease-preventing effects in vivo [2,3,5,7]. In vitro studies have revealed that both the α-LCMs and δ-LCM inhibit the lipopolysaccharide (LPS)-induced production of nitric oxide, 2-cyclooxygenase (COX-2) and interleukin-1β on lipopolysaccharide-activated murine RAW264.7 macrophages. Among the vitamin E metabolites, α-13’-COOH was found to be the most effective in inhibiting both 5-lipoxygenase (5-LOX) in activated human polymorphonuclear leukocytes (PMNL) and leukotriene formation in activated human blood cells at physiologically relevant concentrations. It must be considered that other mediators of the inflammatory network are modulated by α-13’-COOH, such as 12-lipoxygenase (12-LOX) and 1-cyclooxygenase (COX-1) in human platelets, and human leukotriene synthase [3,4,8,10]. Therefore, δ-13′-COOH and α-13′- COOH selectively inhibit 5-LOX activity and the respective biosynthesis of 5-LOX-derived lipid mediators [2,3,4,7]. Unlike the 13′-COOHs, which inhibit 5-LOX and COXs activity, tocopherols do not directly inhibit COX-2 or 5-LOX. Conversely, tocotrienols (e.g., δTE and γTE) can inhibit nuclear factor (NF)-κB activation and have been found to be more potent inhibitors of tumour cell proliferation than the metabolite δTE-13′-COOH. It was documented that the final metabolites of vitamin E, α-CEHC and γ-CEHC, blocked the production of tumour necrosis factor α (TNFα)- or LPS-stimulated PGE2 in mouse microglial cells and rat aortic endothelial cells. The 13′-COOHs inhibit COX-1 and COX-2, and this elucidates the mechanism underlying this action, including the structural characteristics required for inhibiting COXs among different carboxychromanols [3,10,11].

In addition, the effects of the LCMs have been studied in macrophages, phagocytic cells of the immune system. For instance, αT decreased the CD36 expression, a scavenger receptor involved in the oxidized lipoproteins (oxLDL) uptake, thus reducing the differentiation of macrophages into foam cells, known as initiators of the atherosclerotic process [3,11,12,13]. When applied to human macrophages, the alpha-LCMs increased the expression of the scavenger receptor CD36 by roughly four to five-fold. Surprisingly, intracellular oxLDL absorption is reduced, indicating a previously undiscovered independent mechanism that prevents their uptake. The protein PLIN2 (also known as adipophilin) is another player in macrophage foam cell development; it is known to enhance intracellular triglyceride accumulation and creation of cytosolic lipid droplets in macrophages. Stearic acid co-incubation boosted the expression of the lipid droplet-associated protein PLIN2, which protected the cells against stearic acid-induced lipotoxicity. The LCMs then appeared to alter macrophage lipid homeostasis and may operate favourably against atherogenic processes [3,11,12].

The detection of α-13′-COOH studied in human plasma and the modulation of macrophage foam cell formation by α-13′-OH and α-13′-COOH confirmed the first evidence for a new molecular mode of action of αT. Another aspect that was investigated is related to the role of vitamin E metabolites in apoptosis and cell death. Mitochondria-associated cell death was induced in hepatocarcinoma cells (HepG2) upon exposure to micromolar concentrations of α-13’-COOH and δ-13’-COOH by promoting PARP-cleavage, caspase activation, production of reactive oxygen species (ROS), and mitochondrial membrane potential reduction. In addition, the 13’-COOHs showed pro-apoptotic effects in several cell lines, including human THP-1 macrophages and HT-29 colon adenocarcinoma cells. Besides PARP-cleavage, caspases−3 were activated upon treatment of the same LCMs. Mitochondrial-mediated cell death was accompanied by an increase of the intracellular and intra-mitochondrial production of ROS and a collapse of the mitochondrial membrane potential. Pro-apoptotic activity of the 13’-COOHs was also observed in human THP-1 macrophages, as well as glioma, glioblastoma, and HT-29 colon adenocarcinoma cells [3,7,13].

In concluding this section, we specify that from much evidence it seems that the levels of metabolites in serum and their beneficial capacities depend, and can be altered, by various factors that can also be associated with lifestyle and environmental factors, such as: the differences in administration and serum levels of vitamin E metabolites due to age and sex, physical activity and obesity condition, smoking, sleep quality, and alcohol consumption [3,14].

## 4. Vitamin E Effects in Human Health and Diseases

### 4.1. Antioxidant Activity

The term “oxidative stress” (OS) indicates a state of altered balance between antioxidants and oxidants in favour of the latter; the ROS are among the most implicated oxidizing agents. They include the superoxide anion (O2^−•^), the most reactive hydroxyl radical (HO^•^), the peroxyl radical (ROO^•^), and the hydroperoxyl radical (HOO^•^); hydrogen peroxide (H2O2), although it has no unpaired electrons and, therefore, is not a radical, nevertheless can act both as an oxidant and, in the presence of transition metals, can favour the formation of HO^•^. The ROS can represent signals acting as second messengers able to trigger specific cellular responses, both cytotoxic and stress response to high concentrations of pro-oxidants. The consequence of excessive exposure/production of ROS is lipid peroxidation and oxidation of sulfhydryl groups of key proteins (such as enzymes, membrane proteins, etc.). Moreover, the ROS are important mediators of cellular events such as cell proliferation, apoptosis, and can also cause expression of specific factors (including nuclear factor kappa B [NF-κB] and hypoxia-inducible factor-1α [HIF-1α]), and, therefore, lead to abnormalities in cell proliferation and death [2,4,15].

Both physiological and pathological conditions affect the amount of ROS production in mitochondria, but its concentration is always kept under control by the efficient mitochondrial antioxidant system. Generally, when cellular ROS production exceeds the capacity of the mitochondrial antioxidants, the inactivation of enzymes of the Krebs cycle and respiratory chain components can occur. Accordingly, mitochondrial dysfunction is responsible for serious damage to both cell structure and whole body, which result in neurodegenerative and metabolic disease such as aging, dementia, obesity, and type 2 diabetes. The antioxidant system in mitochondria removes excess ROS and repairs damage to biological components and is composed of enzymes and small molecules. Among them, vitamin E is mainly located in mitochondrial membranes and protects them from oxidation by quenching peroxyl radicals. For this reason, the vitamin E supplementation can counteract the mitochondrial OS and prevent harmful consequences [4,6,15].

It is well known that aerobic organisms contain antioxidants (both enzymatic and nonenzymatic), which can maintain redox balance counteracting ROS-induced OS, however under unusual physiological environments, such as those associated with various disease states, antioxidants can be insufficient, thereby initiating lipid peroxidation of cell membranes and damage to proteins and DNA. Furthermore, OS induces various diseases, such as arteriosclerosis, cancer, diabetes, hypertension, metabolic syndrome, and neurological diseases [15]. Whether the antioxidant effect of vitamin E is useful for protecting the body from ROS-induced OS is a matter of controversy [4,6,15].

Vitamin E has been found at the highest concentrations throughout the body and is also present in cell membranes. Its antioxidant power is closely related to the number of methyl groups present in the chroman ring. According to the strength of antioxidant activity, tocopherols are ranked as follows α > β > γ > δ. Particularly, the αT tocopherols are the strongest antioxidants with three methyl groups, while the δTs are the weakest with only one methyl group in the ring. In addition, tocotrienol is weaker than tocopherol [2].

When vitamin E is present, peroxyl radicals react with αT instead of lipids and hydroperoxides, so that the chain reaction which leads to peroxyl radical production is stopped, and further oxidation of membrane PUFAs is prevented. Tocopheroxyl radicals are reduced by vitamin C or glutathione and form tocopherol dimers, which further oxidate or act as pro-oxidants. Therefore, because the activity of signalling enzymes seems to be regulated by the redox state, vitamin E antioxidant activity may regulate the activity of several enzymes involved in signal transduction [3,4,5,16].

Vitamin E inhibits protein kinase C (PKC) activity by increasing PKC--dephosphorylation through the activation of protein phosphatase 2A. This vitamin E-mediated inhibition of PKC has been reported in various cells. Moreover, it has been observed to reduce the proliferation of most immunity cells, vascular smooth muscle cells and decrease superoxide production in neutrophils and macrophages. Therefore, it has been suggested that vitamin E can bind the enzymes involved in the generation of lipid mediators and can be involved in the activity of signal transduction [16].

It has been reported that some cellular proteins, including the tocopherol associated proteins (TAPs), by binding to vitamin E, can prevent its chemical oxidation. The TAPs act as chaperone proteins, which mask the antioxidant hydroxyl residue of vitamin E, and thus the molecule loses its antioxidant capacity. Among plasma proteins, albumin and afamin can also bind vitamin E, protecting it from oxidation. Finally, another proposed mechanism, which inhibits the vitamin E antioxidant activity, is the phosphorylation of hydroxyl groups in the chroman ring [17].

### 4.2. Anti-Inflammatory Activity

The vitamers have anti-inflammatory action by inhibiting a variety of inflammatory mediators. The tocopherols isoforms and tocotrienols suppress COX-mediated production of prostaglandin E2 (PGE2), as well as 5-LOX-mediated production of leukotrienes. The metabolite of vitamin E, 13′-COOH likewise deletes COX and LOX pathways. Eicosanoids from the COX-2 and 5-LOX pathways are associated with cancer pathogenesis. Thus, the suppression of these pathways by vitamin E helps in repressing aggravation of pathogenesis and carcinogenesis. Tocopherols and tocotrienols are found to clamp NF-κB and JAK-STAT3 (kinase-signals transduction and activation of transcription) signalling pathways, mediators of various proinflammatory cytokines, including interleukin-1 (IL-1), interleukin-6 (IL-6), and TNFα. The γTE has the strongest action in this regard. The hypoxia inducible factor-1 (HIF-1)-modulated inflammatory pathway is additionally hindered by vitamin E. Peroxisome proliferator activated receptors (PPAR-α, γ, and δ), are ligand-mediated transcription factors that tweak inflammatory pathways by inhibiting COX. The PPARs are also connected with various pathways like PI3k-Akt and NF-κB signalling. In in vitro and in vivo studies, tocopherols have been shown to announce PPARγ in various cell lines, suppress inflammation, as well as restrain cell cycle progression and actuate apoptosis [3,7,14].

Both OS and systemic inflammation can be influenced by nutrition, so that excessive energy intake, and/or physical inactivity can contribute to pro-inflammatory cytokine secretion. Among other cell types, lipid-laden macrophages and the sub-endothelial region of the artery wall are involved in inflammatory processes. The C-reactive protein (CRP), IL-1, IL-6, IL-8, IL-1, and TNFα are the major mediators of the development of cardio-vascular disease (CVD). The expression of those mediators is also thought to be potentially pro-inflammatory, and it may be related to the severity of CVD [12,18]. These data suggest a link between OS and the development of atherosclerosis. Nitric oxide synthase (NOS), oxidase enzymes, lipoxygenase, uncoupled endothelial nitric oxide synthase, and the mitochondrial respiratory chain through a one-electron reduction of molecular oxygen are some of the additional routes via which ROS and reactive nitrogen species (RNS) are primarily formed. Diverse networks, including catalase, glutathione peroxidase, and superoxide dismutase, among others, control the inhibition and degradation of ROS and RNS. An overload of ROS and RNS causes OS, which promotes DNA damage, endoplasmic reticulum stress, endothelial dysfunction, and increased amounts of oxidized LDL in cells (oxLDL). The production of inflammatory cytokines, like interleukins (IL-6, IL-8), TNFα, and MCP-1, as well as adhesion molecules like intercellular adhesion molecule 1 (sICAM-1) and the vascular cell adhesion molecule, is also raised when ROS are present. This directly affects plaque development and endothelial function (sVCAM-1). There is a significant production of inflammatory cytokines and inducible nitric oxide synthase at the same time as transcription factors, primarily nuclear NF- and nuclear factor (erythroid-derived 2)-like 2 (Nrf2), are activated. Because of its potent vasodilator activity and anti-platelet aggregation, NO has significant anti-inflammatory, antihypertensive, and antithrombotic properties. As a result, a reduction in eNOS NO production leads to endothelial dysfunction, whereas an increase in iNOS NO production may trigger pro-inflammatory and pro-atherogenic factors [6,7,19].

Several research studies have indicated that vitamin E exhibits various potentially beneficial effects on human health, such as anti-allergic [16,18], anti-atherogenic and anti-cardiovascular [6,7,12], anti-cancer [7,20], anti-diabetic [17], anti-inflammatory [3,4,10], antioxidant [4,10], and neuroprotective and immunomodulatory [15,17] properties. Mainly, the relevant ones will be elaborated on in this review.

It often happens that nutritional deficiencies lead to impaired functionality of the immune system; on the other hand, taking recommended doses of certain nutrients can restore or increase immune system function. One of the most effective nutrients able to modulate immune function is vitamin E. This effect is partially due to its ability to prevent oxidation of polyunsaturated fatty acids, which are particularly abundant in membranes of immune cells and are constantly subject to oxidative damage related to high immune cell metabolism and pathogen removal mechanisms [7,10,16,18].

Vitamin E status, influenced by nutritional intake and supplementation, can regulate the immune system and inflammation through several mechanisms, such as the modulation of signal transduction, membrane stability, cell cycle, and production of inflammatory mediators [7,15,18]. The insufficiency of vitamin E results in anaemia, infertility, muscle weakness, and ataxia. Compared with the αT precursor and other vitamers, αT metabolites may be only partially responsible for the effects of αT because of the high rate of metabolization by CYP4F2 [3,4,18]. The vitamin maintenance in the body is under the control of a specific transport protein, αTTP, which selectively enriches only αT. The absence or mutation of αTTP results in a neurological disease known as Ataxia with vitamin E Deficiency (AVED) [3,18].

### 4.3. Interaction of Vitamin E and Gut Microbiota

The human intestine hosts trillions of microbial cells collectively forming a complex microbial community known as “gut microbiota” (GM), which develops over the course of host infancy to reach its adult form. Everyone can be considered as an environment colonized by microbial communities with different rules, able to set a complex diversity.

In this contest, the GM becomes an indispensable tool, which contributes to the correct interaction of host/environment [21,22,23,24,25,26].

The gut microbiota consists of a huge variety of organisms, including fungi, archaea, viruses, protists, and bacteria [22]. Gut microbiota bacteria may belong to any of the four dominant phyla: Firmicutes, Bacteroides, Proteobacteria and Actinobacteria, and the complex relationships that are created among them and with the human host range from symbiosis to parasitism [21,22,23,25,26].

Gut microbiota development and maturation represent a dynamic and non-random process. Microbial colonization of the human gut is believed to be responsible for the concurrent programming of our immune system and the simultaneous development of the intestinal tract and associated metabolism [21,22,23,24]. To orchestrate these physiological processes a continuous dialogue between the microbiota and the host must occur. Several factors can modify the microbiota balance, causing a so-called state of dysbiosis. Intestinal dysbiosis may interfere with the correct dialogue with the host and this, in turn, can cause long-lasting effects and health disorders. Indeed, dysbiosis is usually associated with long-term consequences leading to disorders or diseases, including obesity, diabetes, and inflammatory bowel disease (IBD) [21,22,23,24,25,26,27,28,29].

The composition of gastrointestinal microbiota may be influenced by several environmental parameters including pH, temperature, nutrients, oxygen levels/redox state, and these conditions may allow the proliferation and prevalence of some microbial populations which, while interacting with the surrounding environment, including the human host, exert different activities. Several critical roles are carried out by diverse members of the human gut microbiota maintaining human health by assisting food digestion, to liberate nutrients that would otherwise be inaccessible to the host, and/or by promoting host cell differentiation, by stimulating/modulating the immune system, and protecting the host from colonization of pathogens. For example, it has been shown (in both mice and humans) that the gut microbiota is able not only to influence lipid metabolism, but also to regulate lipid levels in the blood and tissues [21,22,23,24,26,27].

One of the exposure factors that mainly affects the gut microbial ecosystem, is the use of antibiotics that can be responsible for the disruption of intestinal homeostasis leading to a higher relative abundance of Proteobacteria and a concomitant reduction in the abundance of the Bifidobacterium family. Because of this antibiotics induced-dysbiosis, the host metabolism is altered with long-term metabolic impacts and the occurrence of chronic disease including, insulin resistance, type 2 diabetes, obesity, and liver pathologies [21,22,26,27]. Another important role of GM on human health is the demonstration that, as with other fat-soluble vitamins, catabolism and excretion of vitamin E are also influenced by GM and this underlines another aspect of the repercussions that antibiotic therapy can have [26].

Several aspects of fundamental importance for the absorption, metabolism and production of energy in the human host have to be considered: thanks to the ability to stimulate the turn-over of intestinal epithelial cells and the production of the mucus layer, the microbiota behaves as an effective intestinal barrier that opposes the entry of parasites, viruses or bacteria; the end-products of microbiota metabolism are predominantly short-chain fatty acids (SCFAs), which can be used both as energy substrates for intestine cells, and also as signals for communication between the host’s gut and other organs. In fact, SCFAs can activate receptors present in several tissues including adipose tissue, immune system, pancreas, heart, and skeletal muscles, and can also represent valid energy substrates used in pathways including lipogenesis, gluconeogenesis, and cholesterol synthesis [21,22,23,25,26,29].

Recent literature data describes a cross-interaction between Vitamin E and microbiota. Vitamin E can regulate the gut microbiota both directly or indirectly by modifying the immune system and the metabolism of bacteria, which in turn affect the microbial proliferation. Concurrently, the microbial ecosystem can affect several aspects of the metabolism of vitamin E and its bioactive metabolites. An in vitro study [28] showed that vitamins E, A, C, B2 and D can induce a pronounced effect on SCFA. In addition, vitamin E consumption affects the composition of Proteobacteria [30,31]. Recently in a study on rats supplemented with vitamin E and treated with antibiotics, it was observed that perturbation of gut microbiota was involved in the intestinal metabolism of vitamin E mediated by CytP450, thus affecting the levels of circulating Vitamin E and its metabolites [32].

Another link between gut microbiota and both vitamin E and other fat-soluble vitamins can be found in the onset of Metabolic Syndrome (MetS), characterized by the presence of multiple cardiometabolic risk factors, including oxidative stress and inflammation [23,25,31]. It is well known that LPS triggers intestinal and hepatic inflammation as well as oxidative imbalance, which causes lipid oxidation and the consequent depletion of vitamin E amount. Moreover, MetS is associated with lipid disorders and low serum levels of fat-soluble vitamins (A, D, E, K) [24,26,33]. This might lead to metabolic dysfunction that impairs vitamin E trafficking through a mechanism involving the gut.

**Figure 2:**
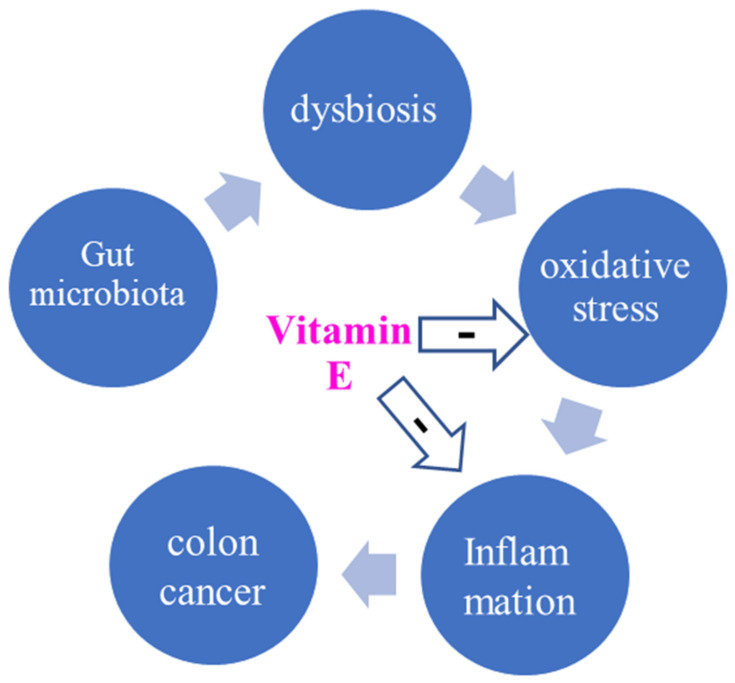
Microbiota-colon cancer loop: the probable modulating effect of Vitamin E.

Diseases linked to dyslipidaemia, such as non-alcoholic liver disease and atherosclerosis, celiac disease, colorectal cancer (CRC), and extra-intestinal disorders, are associated with changes in the gut microbiota profile, and Vitamin C and E showed a direct tumour-suppressing effect on CRC cell lines (Figure 2) [21,22,29].

Studies carried out both on animals and humans have suggested that increased intake of vitamin E may improve immune and inflammatory responses and may be associated with a reduced risk of infectious disease [26,31]. Another study showed that the composition but not the abundance of gut microbiota was regulated by vitamin E δTE (δTE) and its metabolite δTE-13′-carboxychromanol (δTE-13′). Both δTE and δTE-13′ enhanced potentially beneficial *Lactococcus* and *Bacteroides* populations in mice models [3,34,35]. Interestingly, vitamins, not only vitamin E, can affect the GM and host health by affecting the gut immune system, the intestinal epithelial barrier or through direct effects on the GM and subsequently on gut immune and epithelial barrier [22,26,28]. In human and animal studies, vitamins such as vitamin E [30], C, D or A, have been reported to have a modulatory effect on the microbiota [23,26,28]. Conversely, literature supports the notion that GM influences intestinal absorption and post-adsorption metabolism of fat-soluble vitamins [26,31]. Vitamin A plays important roles on the intestinal immunity via interactions with the GM in different regions of the human gut and, vice versa, the microbiota affects retinoid biosynthesis [26,28]. Vitamin D is reported to influence intestinal immunity either by direct or indirect (GM-mediated) processes [25,26]. Vitamin K is reported to play a key role in intestinal homeostasis promoting the selective growth of symbionts. Moreover, it is involved in the respiratory electron transport chains of prokaryotes, also providing antioxidant activity and protection of cell membranes against lipid peroxidation [22,26].

### 4.4. Vitamin E and Cardiovascular Diseases

Vitamin E has demonstrated protective effects by reducing atherosclerotic plaque formation in animal models; dietary consumption of foods rich in vitamin E has been associated with lower cardiovascular disease (CVD) outcomes both in humans and animal models. On the other hand, the results regarding the ability of vitamin E to protect against CVD are controversial [4,6,7,12,13,36]. Cardiovascular diseases are among the primary causes of adult mortality worldwide, although controlling disease risk remains difficult. Cardiovascular diseases refer to a range of heart and blood vessel disorders such as hypertension, stroke, atherosclerosis, peripheral artery disease, and vein diseases. Cardiovascular disease is also linked to poor dietary habits; for example, CVD is frequently associated with various comorbidities, such as obesity, diabetes, hypertension, or dyslipidaemia. A substantial body of scientific research suggests that diet may be the most important factor in preventing CVD mortality and may potentially reverse heart disease. This review focuses on two key CVD-related pathologies: atherosclerosis and ischemia.

Atherosclerosis is an inflammatory disease, characterized by the production of inflammatory cytokines and decreased nitric oxide levels, which lead to vasoconstriction and compromised vascular structure, that contributes to incidence and mortality of CVD, and which the application of vitamin E can be a step to halt the process [3,4,7,12,13,37]. Early stages of atherosclerosis involve the internalization of lipids in the intima, mainly low-density lipoproteins (LDL), which is translated to endothelial dysfunction (nevertheless, not all individuals who develop CVD present these established risk factors, and thus complicates early disease detection) [6,12,37]. The disruption of the endothelial function promotes the inflammatory response, thrombus formation, and multiple pathological consequences, such as stenosis, rupture, or haemorrhage [12,36,37]. The infiltration of LDL particles into the extracellular matrix boosts the inflammatory response, while circulating monocytes adhere to the endothelium and change into macrophages that infiltrate the sub-endothelial region. The ROS and enzymes generated by inflammatory cells are capable of oxidizing LDL particles. The differentiation of macrophages into foam cells with the scavenger receptor CD36, which is responsible for the absorption of oxidized lipoproteins (oxLDL) into the cell and their subsequent transformation into foam cells, is a critical event during atherogenesis. Vitamin E, through its T and LMCs, lowered CD36 expression in both antioxidative and non-antioxidative ways [3,7,12,13,37]. Furthermore, αT or mixed tocopherols elevated blood pressure without altering cytokines or endothelial-dependent or independent vasodilation. The γT’s potential modulatory effects on inflammation and OS have been studied in healthy persons. For instance, severe exercise-induced coagulation and aggregation of platelets were reduced by supplementation with a blend rich in αT or γT tocopherols [4,12,37]. Additionally, a study conducted on mice showed that the concomitant administration of vitamin E and coenzyme Q was able to reduce the progression of atherosclerosis [12]. In the following, endothelial dysfunction enhances platelet adhesion, which secretes chemotactic substances and growth factors, promoting plaque progression [12,20,37]. If the progression of lipid accumulation persists, foam cells and macrophages apoptosis is induced with pro-thrombotic molecules secretion. Atherosclerotic plaque progression and plaque disruption, promoted by pro-thrombotic agents, initiate platelet activation and aggregation, which lead to the coagulation cascade and, consequently, thrombus formation [12]. Lipid oxidation, whether enzymatic or non-enzymatic, leads the isomerization of fatty acid cis-double bonds to trans-double bonds. By reversible addition to the double bond, the nitrogen dioxide radical drives isomerization of cis-fatty acids to trans-fatty acids. The cis-trans isomerization of phospholipids’ fatty acid moiety causes membrane structural and dynamic disorder, resulting in a shift in membrane permeability. Trans fatty acid consumption in the diet has been related to several negative health impacts, including an increased risk of cardiovascular disease [3,4,6,12,37]. The major lipid oxidation products observed in advanced atherosclerotic lesions and ischemia/reperfusion were cholesterol esters and hydroxyoctadecadienoic acid (HODE). In the hearts of α-TOH-treated mice, there was a substantial reduction in oxidized HODE when compared to controls. With a diminution in oxidized lipids, researchers have demonstrated that αT administration causes an anti-oxidative response in the heart following ischemia/reperfusion (I/R) damage. In this study, researchers were also prompted by a protective impact of αT on mitochondrial integrity, which protected heart function and increased recovery after I/R [36]. Long-chain carboxychromanols 13′-COOHs but also αT have been shown to inhibit COXs and 5-LOX [3,4,7,12,37]. The involvement of the nucleotide-binding domain and leucine-rich repeat pyrin domain inflammasome (NLRP3) in many chronic diseases, including CVDs, is well known. Various vitamin E vitamers, as well as some LCMs, have been shown to inhibit this pathway. Targeting the arachidonic acid pathway, which is known to interact with NLRP3, may be a possible mechanism of inhibition mediated by vitamin E metabolites [12,36,37]. The clinical manifestations of advanced atherosclerosis are coronary heart disease, ischemic stroke, peripheral artery disease, heart failure, or sudden death [3,37]. Ischemic heart disease is a kind of CVD that is associated with pathological conditions such as myocardial infarction (MI). This I/R damage is characterized by necrosis of cardiac tissue induced by a combination of severe inflammatory and OS. A recent study established the ability of αT to retain heart function following acute MI in a murine model of myocardial I/R [36,37], so αT reduces oxidation of lipids in the myocardium independent of neutral lipid accumulation and systemic lipid profile. In addition, the role of vitamin E in other cardiovascular disorders, including ischemic heart disease and heart failure, has been tested with several in vitro models. Daily dosage of a nutrient mixture, consisting of vitamin E, vitamin C, and L-arginine, was shown to reduce risk factors in CVD. This study also showed a reduction in ischemic heart disease risk among women using vitamin E, vitamin C, and/or vitamin A without multivitamins or plus multivitamins cardioprotective role [12,36]. Previous studies have demonstrated that treatment with αT reduced the risk of myocardial infarction in patients with coronary atherosclerosis [12,36]. A prevention with antioxidants of cardiovascular disease in end-stage renal disease study showed that αT administration significantly reduced the composite endpoint of myocardial infarction (fatal and non- fatal) ischemic stroke in patients with chronic kidney disease. Heart failure is yet another type of CVD that is defined by a depleted metabolic energy reserve and the activation of multiple molecular processes that contribute to cardiac hypertrophy, inflammation, fibrosis, and apoptosis. Heart failure can occur under extreme stress conditions due to a decrease of cardiomyocyte cell function, which can promote apoptotic or necrotic death processes. Vitamin E appears to be effective in reducing apoptotic activity. Tocotrienols have been identified as a group of cardioprotective molecules due to their capacity to activate proteasomes and improve myocardial function [12,36]. This report can suggest that a vitamin E therapy/supplementation could compensate for I/R injury and atherosclerosis by an adequate anti-oxidative and anti-inflammatory support. However, there remains a need to focus on in vivo data for humans and whether vitamin E exhibits modes of action different from those found in vitro.

### 4.5. Vitamin E and Diabetes Mellitus

The term diabetes mellitus (DM) refers to a series of heterogeneous pathologies characterized by chronic hyperglycaemia. Within this group, three main types of diabetes can be identified: type 1 diabetes (T1DM), type 2 diabetes (T2DM), and gestational diabetes mellitus (GDM). It is possible to identify other types of diabetes, defined as “other specific types of diabetes”, due to factors like genetic defects of both the β-cell and insulin metabolism, diseases of the exocrine pancreas, endocrinopathies, infections, drugs, and chemicals [38]. The World Health Organization (WHO) reported that more than 95% of diabetes cases are type 2 and the global prevalence of diabetes rose from 47% in 1980 to 85% in 2014 [39]. In the onset of T2DM, a complex multifactorial disease, several factors are involved such as genetic factors, vitamin deficiencies, gut microbiota composition, lifestyle, and excessive body weight [40]. A complex vicious circle between T2DM and OS sustains the occurrence and development of DM. Notably, OS plays an important role in the pathogenesis of this disease by damaging pancreatic β-cells, reducing the expression of glucose transporters GLUT-4, causing mitochondrial dysfunction, and altering the normal insulin signal transduction as well as reducing the antioxidative defence system of the body [41].

As widely reported in the literature, low concentrations of antioxidants increase the risk of diabetes complications (e.g., coronary artery and cardiovascular diseases, retinopathy, and nephropathy), which are the leading causes of morbidity and mortality worldwide. Some studies have shown that the intake of antioxidants by diet can prevent or retard the DM onset [42].

Kataja-Tuomola et al. [43] conducted a study on male smokers aged 50 to 69 and found no relationship between αT serum levels and T2DM. Concurrently, the vitamin E supplementation (DL-α-tocopheryl acetate, 50 mg/day) did not reduce the incidence of the disease. However, as suggested by the authors, these results cannot be extended to other populations not included in this study. Previously, Mayer-Davis et al. [44] found that in vitamin E supplement nonusers, the individuals who developed diabetes had lower αT plasma levels. However, no protective effect of vitamin E supplementation was found. Similarly, Liu et al. [45] demonstrated no preventive effect against diabetes after the administration of 600 IU of vitamin E in women over the age of 45, initially not affected by diabetes.

Numerous studies in the literature have evaluated the ability of vitamin E to counteract the side effects of diabetes, thus helping to control the disease. The increase in blood glucose levels due to diabetes causes an overproduction of ROS, which is responsible for many of the complications associated with this disease [46]. Bearing in mind this evidence, Pazdro et al. [46] reported that vitamin E would be able to protect against some of these diabetes-associated complications due to its marked antioxidant activity. Vitamin E is not only able to act as a primary antioxidant, reducing lipid peroxidation and damage to DNA and proteins induced by OS in diabetic patients, but it promotes the increase of endogenous antioxidant defences. Based on these properties, vitamin E may be able to protect the pancreas, the kidney, the eyes, and the nervous systemin in animal models [46]. However, Pazdro et al. [46] also reported that conflicting results were obtained when tests were carried out on humans.

A direct correlation between diabetes and atherosclerosis is well known, and is associated with factors such as dyslipidaemia, OS, inflammation, and hyperglycaemia. One of the pathways involved in the development of atherosclerosis in diabetic patients is regulated by the activation of protein kinase C (PKC) induced by OS and the high glucose intake in the endothelial cells, which in turn causes an overproduction of diacylglycerol (DAG), a PKC activator [47]. The PKC contributes in various ways to the pathogenesis of atherosclerosis, such as reducing NO production and increasing macrophage activation [48]. Bursell and King [49] suggested a possible protective role of vitamin E against diabetes-induced atherosclerosis mediated by the inhibition of the DAG-PKC pathway. Derevaj and Jialal [50] reported a similar protective effect of αT supplementation for three months (1200 IU/die), which reduced C-reactive protein (CRP) and IL-6 plasma levels. More recently, Xu et al. [51] analysed in a review the possible preventive action of antioxidant molecules, in particular vitamins C and E, against the cardiovascular complications of diabetes. For atherosclerosis, specifically, they reported that, despite the promising results of experimental and epidemiological studies, many of the clinical trials did not show protective actions. The authors suggested that this inconsistency may be due to inadequate monitoring of patients’ OS, too low doses of vitamins during treatment, and the start of supplementation when the disease was already advanced. Furthermore, they reported that supplementation was most effective when a combination of vitamin C and vitamin E was given, and that the administration is useful in preventing the onset of atherosclerosis but is ineffective in reversing it [51].

Nephropathy represents another of the most common and serious complications associated with diabetes. In its pathogenesis, a key role is played by OS, but recently the importance of inflammation in the onset of nephropathy has also been highlighted [52]. Khatami et al. [53] evaluated the effect of administering high doses of vitamin E for 12 weeks on some parameters related to renal damage, inflammation, and OS in patients with diabetic nephropathy. Their results showed a significant decrease in the levels of urinary protein and protein-to-creatinine ratio (PCR), TNFα, matrix metalloproteinase-2 (MMP-2), matrix metalloproteinase-9 (MMP-9), malondialdehyde (MDA), advanced glycation end products (AGE), and insulin concentrations in serum. However, no significant differences were observed for other biomarkers, fasting glucose levels, and insulin resistance. Tan et al. [54] conducted a clinical trial that showed how a tocotrienol-rich vitamin E supplement, namely Tocovid, was able to significantly improve renal function in patients with diabetic-induced nephropathy, whilst it had no effect on the reduction of blood glucose levels.

Shi et al. [55] suggested a possible protective role of vitamin E against diabetic retinopathy, due to its antioxidant properties and its capacity to reduce blood pressure abnormalities and to improve retinal blood flow. They also proposed that a combination of vitamin E and vitamin C could be more beneficial. Furthermore, Tabatabaei-Malazy et al. [56] reported that several studies have shown low serum levels of vitamin E in patients with diabetic retinopathy. Considering its antioxidant properties and its ability to reduce leukocyte adhesion in retinal vessels, the authors suggested that vitamin E may have protective properties against diabetic retinopathy, in particular in preventing or delaying its onset, especially when in association with vitamin C. Similar conclusions have been reached by She et al. [57], who conducted a study on patients suffering from T2DM, finding that vitamin E appears to have protective effects against diabetic retinopathy. More recently, Ho et al. [58] found that a daily supplementation of Tocovid (200 mg, twice daily, for 12 months) had beneficial effects on patients with diabetic retinopathy.

### 4.6. Vitamin E and Asthma

Asthma, a major non-communicable disease, encompasses a range of endotypes, with different phenotypes, which share symptoms such as wheezing, shortness of breath, cough, chest tightness, and airway obstruction. Based on the pathophysiological mechanisms, it is possible to identify two major endotypes: T2-high and non-T2-high. Subjects with the T2-high endotype often have a dysregulated epithelial barrier that allows the allergens to pass through, leading to the release of alarmins and the consequent differentiation of T-cells into Th2 cells. Then, Th2 cells cause the activation of B cells via IL-4, which differentiate into IgE producing cells. Differently, in the non-T2-high endotype, a neutrophil- and paucigranulocytic-mediated inflammation and the activation of Th1 and/or Th17 cells were observed [59]. Immune cells, inflammation and OS are positively associated with the occurrence of asthma. In particular, the overproduction of ROS can result in tissue damage of the airways of asthmatic patients mediated by generation of eosinophil extracellular traps [60]. Asthma affects people of all ages, but it is most common among children [61].

Many studies have investigated the correlation between vitamin E intake in pregnancy and the likelihood of asthma in children. Wu et al. [61] conducted a meta-analysis considering nineteen studies and found that maternal intake of vitamin E was protective against childhood asthma development. Similarly, Devereux et al. [62] showed that the intake of vitamins D and E was inversely proportional to the risk of developing childhood asthma before the age of 15.

Alwarith et al. [63] suggested a possible preventive role of vitamin E due to its antioxidant and anti-inflammatory properties, preventing endothelial damage and reducing leukocyte recruitment. Additionally, Lewis et al. [19] reported that vitamin E is effective in the prevention of asthma due to its anti-inflammatory and immunomodulatory properties. Indeed, the authors reported studies that not only confirmed the association between maternal intake of vitamin E and prevention of asthma, but also highlighted an inverse correlation between serum levels of vitamin E in children and the onset of asthma. Interestingly, they also explained that this relationship is much less clear when adult-onset asthma is considered, but specifying that, regardless of the onset of the disease, the intake of vitamin E was able to reduce its severity.

However, the literature on the subject is sometimes contradictory and, in this regard, Strait and Camargo [64] tried to give a possible explanation on the different results obtained. The authors reported various studies on the relationship between vitamin E and asthma, concluding that supplementation of vitamin E may be effective in preventing this pathology. They suggested that the contradictory results may be due to the heterogeneity of the disease and the different vitamers of vitamin E, each with different biological properties. Furthermore, as regards integration during pregnancy, the authors hypothesized the presence of a time window during which the integration itself could be effective, however reporting that the optimal timing was not clear.

### 4.7. Vitamin E and Cancer

Although the scientific literature on the relationship between vitamin E and cancer is particularly extensive, it is beyond the scope of the present paper to provide an exhaustive and comprehensive overview on this topic. Therefore, we focused on the four most common cancers according to the WHO classification in 2020, in order: breast cancer (2.26 million cases), lung cancer (2.21 million cases), colorectal cancer (1.93 million cases), and prostate cancer (1.41 million cases) [65].

Besides being the most common cancer, breast cancer is one of those with the highest mortality rate [65]. Various types of classification can be used to distinguish the different types of breast cancers. Traditional categories include subdivision based on parameters such as histologic type, grading, immunophenotype, tumour size, nodal status, and distant metastasis staging. As regards the immunophenotype, a classification can be made considering the expression of a nuclear sex steroid receptor, the Estrogen Receptor (ER). Most ER+ tumours are also Progesterone Receptor (PR) positive, and this kind of tumour is usually less aggressive [66].

Xin et al. [67] investigated the relationship between vitamin E circulating levels and incidence of ten cancers. For breast cancer, they found that vitamin E levels were associated with a reduced breast cancer risk, but due to the low statistical significance of the result, the authors reported that it was not possible to establish a causal relationship between the two factors. Similarly, Fernandez-Lazaro et al. [68] investigated whether antioxidants may have a protective role against breast cancer, but no relationship was found except for vitamin E dietary intake in postmenopausal women. Interestingly, Playdon et al. [69] demonstrated that various vitamin E vitamers were differently associated with ER+ breast cancer risk. In particular, they reported an inverse correlation with serum αT and a positive correlation with serum ẟT and conjugated γT.

As regards lung cancer, it is the one with the highest mortality in 2020 according to WHO [65]. Lung tumours can be divided into two histological categories: non-small cell lung carcinoma (NSCLC) and small-cell lung carcinoma (SCLC). The NSLCs are the most frequent, accounting for 80–85% of lung cancers, and include adenocarcinoma, squamous cell carcinoma, and large cell carcinomas [70].

Knekt et al. [71] conducted a study on the relationship between αT serum levels and cancer. They found that cancer patients had, on average, αT serum levels 3% lower than the control and that people with low serum vitamin E were 1.5 times more likely to develop cancer. However, the association differs from cancer to cancer according to the tumour site. Accordingly, it was stronger, for instance, in some gastrointestinal cancers (like stomach and pancreas), while no association was found with lung cancer. This result is in accordance with the study by Heinonen et al. [72] that reported no reduction in incidence and mortality in lung cancer patients who had received vitamin E supplementation. More recently, Huang et al. [73] conducted a prospective study on male smokers, finding that serum αT levels and lung cancer risk were inversely associated, in particular for squamous cell carcinoma. This clinical trial also included vitamin E supplementation (DL-α-tocopheryl-acetate, 50 mg/day) but, interestingly, the authors found no correlation between increase in serum vitamin E levels after three years and lung cancer risk, except for subjects who initially had serum vitamin E concentration <10.5 mg/L and who had a great increase through diet and without supplementation. A significant reduction in the risk of lung cancer was observed for these men suggesting that a healthy lifestyle, including proper nutrition, may be more effective than supplementation in the prevention of this disease.

A meta-analysis by Dong et al. [74] evaluated the correlation between colorectal cancer and serum vitamin E levels. They found in a Caucasian population, significantly lower serum levels of vitamin E in subjects with colorectal cancer than in the healthy population, while no difference was found in an Asian population. The authors concluded that low serum vitamin E levels may be a risk factor for colorectal cancer. On the other hand, Chapelle et al. [75], in a review, concluded that vitamin E supplementation had no effect in preventing colorectal cancer. Similarly, Yang et al. [76] reported some studies showing no relationship between vitamin E intake and colorectal cancer risk, except for that conducted by Luo et al. [77], who found an inverse association between vitamin E intake and colorectal cancer risk, but no correlation between the disease and serum αT levels.

Prostate cancer is the second most frequent tumour in the male population [78], but the data in literature for it are also conflicting. Cui et al. [79] conducted a meta-analysis on nine studies evaluating the relationship between αT and γT levels and prostate cancer. Their results suggested that αT was inversely associated with the risk of prostate cancer. In fact, the authors reported that every 25 mg/mL increment of blood αT level led to a 21% decrease in the risk of developing the disease. On the other hand, for γT, this correlation did not seem to exist, but the authors suggested that the preventive potential of this molecule for prostate cancer may be masked by the high levels of αT. Conversely, Lippman et al. [80] found no evidence of any preventive potential of αT in men who had received 400 IU/day of all-rac-α-tocopheryl acetate. The authors suggested that the absence of a protective effect may be due to the high dose of αT used in the study, negatively affecting the action of γT taken in through diet.

The main effects of vitamin E and its metabolites discussed in this chapter are summarized in the Table 1.

## 5. Toxicity and Health Risk of Vitamin E

The toxicity of Vitamin E is rarely observed as the pharmacokinetic process allows a rapid excretion of Vitamin E and its metabolites, thus reducing the level of circulating vitamers [81]. Although there is no evidence for Vitamin E side effects from dietary intake, vitamin supplementation, at more than 1100 IU/day for weeks to months [82,83], was mainly associated with haemorrhagic effects and impaired coagulation [84], and in vitro inhibition of platelet aggregation [85]. Other side effects have been reported, including muscle weakness, fatigue, gastrointestinal manifestations, emotional disorder, and thyroid problems. These complications can be overcome by interrupting vitamin E supplementation [84].

In addition, an increased all cause-mortality risk was described in two meta-analyses of randomized trials [86,87]. However, those studies have some bias, because they included middle-aged or older participants with chronic diseases or related risk factors, thereby no convincing evidence can be found showing potential adverse effects of high-dose vitamin E supplements.

Recently, the SELECT trial demonstrated that 400 IU/day of dl-alpha-tocopheryl acetate may increase the risk of prostate cancer [88] even if follow-up studies are ongoing to address this issue.

Finally, long-term vitamin E supplementation did not prevent cancer or major cardiovascular events but may increase the risk for heart failure in patients with vascular disease or diabetes mellitus [89].

## 6. Conclusions

The antioxidant and anti-inflammatory effects of vitamin E are well known and extensively studied. However, the data in literature are conflicting regarding the beneficial effects of this vitamin against chronic diseases. A possible explanation could derive from the high heterogeneity of many pathologies, as well as the presence of different isoforms of vitamin E, which can exert different biological activities, and different metabolic activity from individual to individual. Furthermore, the antioxidant systems of the human organism are multiple and largely interconnected, for this reason the integration of vitamin E alone may not be sufficient to restore the physiological redox balance. Moreover, the relevant role played by the intestine and gut microbiota in the processes of absorption, transformation, and subsequent transport of the vitamers of this fat-soluble vitamin should not be overlooked.

## Figures and Tables

**Table 1:** Summary of the known and potential vitamin E effects on some non-communicable pathological conditions.

Pathological Conditions	Reported Effect	References
Oxidative stress	Antioxidant by scavenging free radicals, especially peroxyl radicals, and singlet oxygen.	[2]
	To maintain the integrity of long-chain polyunsaturated fatty acids in the membranes of cells and thus maintain their bioactivity. Maintaining the membranes integrity by inhibiting long-chain polyunsaturated fatty acids oxidation	[10]
	Preserving mitochondrial efficiency	[4,6,15]
Inflammation	Inhibition of inflammation mediators and related enzymes	[3,4,18,19]
Dysbiosis	Modulatory effect on gut microbiota	[30,31]
CVD	Reduction in atherosclerotic plaque formation, CVD outcomes, myocardial infraction, and ischemic stroke risks	[12,36]
	Lowering of CD36 expression	[3,12,37]
	Elevation of blood pressure by αT or mixed tocopherols	[4]
	Reduction in platelets coagulation and aggregation with αT or γT supplementation	[4]
	Reduction in atherosclerosis progression with vitamin E and coenzyme Q supplementation	[4]
	Reduction in oxidized HODE in the hearts of α-TOH-treated mice	[36]
	αT administration causes anti-oxidative response following I/R damage	[36]
	Protective effect of αT on mitochondrial integrity	[12,36]
	Inhibition of COXs and 5-LOX by 13′-COOHs and αT	[3,4,7]
	Reduction in lipids oxidation in the myocardium by αT	[36,37]
	Reduction in CVD risk factors with vitamin E, C and L-arginine	[12]
	Reduction in ischemic heart disease risk using vitamin E, C, and/or vitamin A	[12]
	Reduction in apoptotic activity with vitamin E	[12]
Diabetes	No relationship with αT serum levels	[43]
	Lower αT plasma levels in subjects with T2DM	[44]
	No protective effect of vitamin E supplementation	[43,44,45]
	Protective effect against diabetes-induced complications	[46]
	Protective effect against diabetes-induced atherosclerosis	[49,50]
	Conflicting result about the protective effect against diabetes-induced atherosclerosis	[51]
	Protective effect against diabetes-induced nephropathy	[53,54]
	Protective effect against diabetes-induced retinopathy	[55,56,57,58]
	Lower vitamin E serum levels in people with diabetes-induced retinopathy	[56]
Asthma	Protective effect of vitamin E maternal intake	[19,61,62]
	Protective effect of vitamin E	[19,63,64]
Breast cancer	Possible protective effect of vitamin E	[67]
	Inverse association with breast cancer risk only for vitamin E dietary intake in postmenopausal women	[68]
	Inverse relationship between αT serum levels and ER+ breast cancer risk, but positive relationship with serum ẟT and conjugated γT.	[69]
Lung cancer	No relationship with αT serum levels	[71]
	No protective effect of vitamin E supplementation	[72]
	Inverse relationship between αT serum basal levels and lung cancer risk	[73]
	No protective effect of vitamin E supplementation	[73]
Colorectal cancer	Lower vitamin E plasma levels in subjects with colorectal cancer (for Caucasian population)	[74]
	No protective effect of vitamin E	[75,76]
	Inverse relationship between vitamin E intake and colorectal cancer risk, but no relationship with αT serum levels	[77]
Prostate Cancer	Inverse relationship between αT serum levels and prostate risk, no relationship with γT	[79]
	No protective effect of αT supplementation	[80]

## Data Availability

Not applicable.
